# Dr. Marie Stopes' Utopia

**Published:** 1920-11-06

**Authors:** 


### Dr. Marie Stopes' Utopia.

One of the boldest statements in the book on the Control of Parenthood to which we recently called attention is that made by Dr. Marie Stopes, the well-known writer on the relationships, who, in discussing the imperial and racial aspects of the question of birth control, declares at the outset "the sad features of racial degeneration which assail us on our every side to-day" are nearly all the result of two great wrongs. One is crowding, and the other the "devastating infections" known as venereal diseases. Crowding before birth, she explains, crowding in the womb of the overburdened mother, is at present the greatest of all the natural sources of the dwarfing and stunting of humanity, sapping the resources of the race in every direction, and this will in her opinion for ever remain until humanity takes complete control of its conceptions. Sex disease may—should—be speedily eliminated, but the impulse to overpopulate is an inherent characteristic in untutored humanity. She sets down as axiomatic the views that ante-natal influence through the mother is so certain that not only the bodily condition, but the mental and spiritual outlook, of the mother affects the child she is bearing. What is the effect of the working of this law on the race? "Do we not see it all round us in the bitterness, the hatred, the inhuman virulence of one human being towards another. Such dispositions are the very counterpart of the feelings of outraged horror and revolt which overwhelm the already overburdened mother, when she feels the drag within her of yet another child she does not desire." We are to understand from Dr. Stopes that when once the women of all classes have the fear and dread of undesired maternity removed from them they will be free to put all their delicate strength into creating desired and beautiful children. "And it is on the feet of those children that the race will go forward into the promised land of Utopia." This, the first foundation of Utopia, could be reached, Dr. Stopes is convinced, in her lifetime, had she the power to issue inviolable edicts. That is where the difficulty begins. One can imagine no more hopeless subject about which to try to issue "inviolable edicts."

Professor J. A. Thomson is less imaginative and more ready to compromise in an essay which discusses the question chiefly from the biological point of view. He personally shares the view of Mr. Havelock Ellis that control within limits makes for progress and is likely to continue to do so, being not "race suicide" but race-saving. At the same time he concedes that if the mode of life and thought we are inclined to acquiesce in tends towards a mere national history view of marriage and children we must correct it. "While we must not allow the word 'artificial' to be a bogey, we know that the substitution of mechanical control for moral control can never be regarded with entire equanimity. We must, to save ourselves, cultivate counteractives to mechanisation, for if we lose the chivalry and tenderness of lovers, the joyousness of the springtime of the heart, the adventurousness of early marriage on meagre material resources, and the delight of having children while we are young enough to sympathise with them, we are missing some of the fragrant flowers of life."

It is a pity that the experts seem to be in such complete disagreement upon the vital questions of birth control that for the general public there appears to be no immediate hope of direct and reliable guidance. It cannot be too emphatically insisted upon that without scientific guidance, loudly and plainly given, the evils of half-ignorant birth control on the one hand and an ignorantly unrestricted birth-rate on the other must inevitably shape themselves.

